# Spectroscopic ellipsometry modelling of Cr^+^ implanted copper oxide thin films

**DOI:** 10.1038/s41598-023-49133-x

**Published:** 2023-12-13

**Authors:** K. Ungeheuer, K. W. Marszalek, M. Mitura-Nowak, A. Rydosz

**Affiliations:** 1 Faculty of Computer Science, Electronics and Telecommunications, AGH University of Krakow, 30 Mickiewicza Ave., 30-059 Krakow, Poland; 2 Henryk Niewodniczanski Institute of Nuclear Physics, 152 Walerego Eljasza Radzikowskiego Str., 31-342 Krakow, Poland

**Keywords:** Materials for energy and catalysis, Techniques and instrumentation

## Abstract

In this paper, we present modelling of spectroscopic ellipsometry data. The measured samples are thin films of copper oxides modified with the ion implantation method. The samples were prepared using reactive magnetron sputtering. Thin films of CuO and Cu_4_O_3_ were deposited and subjected to Cr ion implantation with an energy of 15 keV and a dose of 5 × 10^16^ ions/cm^2^. The decrease in crystallinity of the thin film as a result of the implantation was inspected with X-ray diffraction measurements. The implantation of Cr^+^ ions was simulated using the Stopping and Range of Ions in Matter software by Ziegler and Biersack. Ion beam energy of 15 keV was simulated to estimate the distribution of Cr ions in the copper oxides thin films. Optical parameters, such as refractive index, extinction coefficient, and absorption coefficient of the thin films, were investigated with spectroscopic ellipsometry. Multilayered models based on Tauc–Lorentz oscillators were developed for both oxides. Analysis of the optical properties showed that the ion implantation with Cr decreased the absorption of copper oxides thin films and the modelling proved that the material properties of top layers changed the most.

## Introduction

Copper oxides are known semiconductors with a high absorption coefficient. Their optical and photocatalytic properties bring great interest among researchers^1–3^. Although most researchers focus on studies of cupric (CuO) and cuprous (Cu_2_O) oxides, where paramelaconite (Cu_4_O_3_) is not investigated that much, where a good example is that the first application of Cu_4_O_3_ in a solar cell system was published in 2012, while copper oxide Cu_2_O was one of the first photovoltaic materials^4^. Possible applications of copper oxides are in photovoltaics^3,5^, sensors^6,7^, and photocatalysis^8,9^. However, the performance of the material is lower than expected theoretically. For example, copper oxides as photovoltaic absorbers in thin-film solar cells should give energy conversion efficiency of 20% (Cu_2_O) or even 30% (CuO), based on the Shockley-Quisser limit and the value of the energy band gap of the oxides. Meanwhile, the best performances of solar cells with copper oxide as an absorber cannot reach even 10% efficiency^10,11^. Modifications are crucial to improve properties of copper oxides thin films and achieve better performance in photovoltaics^12^ and other applications.

Here we report research with copper oxides modification using the ion implantation method. We chose for our experiment one of well studied oxides—CuO, and Cu_4_O_3_ as the reports on this material are few. We chose Cr as dopant, as it is a transition metal and can improve the electrical properties of copper oxides^13^. In other works, the researchers used Na^14^, Co^15^, Ag, Au, Cr, Pd, Pt, Sb, Si^16^, and Ni^17^ as dopants to modify the properties of copper oxides. Furthermore, Cr has been proved to enhance the sensing performance of copper oxides^18^ and increase the V_OC_ and I_SC_ of a CuO:Cr/p-Si p–n junction, which is favourable in solar cell applications^19^.

Ion implantation is a method of material doping widely used in the semiconductor industry^20,21^. This technique enables us to input dopant atoms to the required depth of the material. The process also induces damage and even amorphization of the target film, thus annealing and recrystallisation of a semiconductor is necessary to achieve desired electrical properties. Ion implantation was also used as a modification method for copper oxides, for example, with N ions to introduce a phase change^22^, or the tune electrical and optical properties^23^. CuO nanowires were modified with Fe and N ion irradiation by Sisman et al.^24^ where energy of 60 and 100 keV was used, with a fluence of 1 × 10^13^ and 2.5 × 10^16^ cm^−2^. The thin film consisting of nanowires was about 1 μm thick. The electrical and sensing properties were improved, though the X-ray diffraction measurements did not allow to see any structural changes caused by implantation, only using the X-ray photoelectron spectroscopy they could state phase changes after implantation to Cu_2_O and Cu(OH)_2_. Another utilization of ion implantation was performed by Shi et al.^25^, who used argon ions to introduce defects into CuO to study the magnetic properties of the material. High energy 2 MeV Ar ions with doses of 1 × 10^15^ and 5 × 10^15^ cm^−2^ produced an increased amount of the Cu vacancies and cause a phase change of CuO into Cu_2_O. Yadav et al.^26^ implanted CuO thin films of 220 nm thickness with Ag ions of 30 keV energy and doses between 1 × 10^14^ and 1 × 10^16^ cm^−2^. They found that with higher dose the crystallinity of material decreased, and they also noticed a phase change—into Cu_2_O and even Cu for the highest implantation dose. Atomic force microscopy showed an increase in roughness after implantation. The energy band gap of CuO determined with diffused reflectance spectroscopy was reduced with implantation. However, no reports have been published on the use of ion implantation to modify Cu_4_O_3_ thin films.

To study the influence of implantation, we characterised the structural and optical properties of the thin films with X-ray diffraction (XRD) and spectroscopic ellipsometry (SE), respectively. To analyse the data obtained with the SE method, a model of material dielectric function is required. Here we designed a model based on the Tauc–Lorentz oscillators and information on energy transitions that occur in copper oxides. In a review of copper oxides Meyer et al.^4^ studied the dielectric function of these materials, and provided ε_2_ transition energies. The models used in this work were created on the basis of that information.

We additionally performed simulations to find how deep ions induce changes in the material, for this purpose we used software of Stopping and Range of Ions in Matter (SRIM)^27^. The calculation gives many items of information as output, enabling us to study how ions interact with the target. The calculations of this program are based on two main effects, the electrical and nuclear stopping powers, which correspond to the interaction of the implanted ions with the bound electrons and nuclei of the target atoms, respectively. The simulation assumes that the material is amorphous and therefore channelling effects are not considered. The software requires as input parameters the composition of the target material, its thickness and density, as well as information about the implantation beam: type of ion, energy, and angle of incidence. The package returns information about the range and straggling of implanted atoms, the damage caused by recoil cascades, energy absorption, and ionisation of target atoms.

## Material and methods

### Film deposition

The cupric oxide and paramelaconite thin films were deposited with the DC reactive magnetron sputtering method. The substrate used was monocrystalline silicon to perform characterisation of deposited films with X-ray diffraction and spectroscopic ellipsometry. Before deposition, the substrates were cleaned with warm water and soap, and then submerged in isopropanol for 20 min of an ultrasonic bath. For deposition of thin films we used a 99.95% purity copper target from Kurt J. Lesker; the distance between the substrates and the target was 5 cm. During the sputtering process, the substrates were heated to 150 °C. Before the deposition itself, a pre-sputtering process was performed, with the aim of clearing the target and creating ions needed for the sputtering process. The pre-sputtering lasted for 10 min in Ar and then for 20 min in working gas. For CuO deposition, the working pressure was 2.00 Pa and the atmosphere consisted of only O_2_ with gas flow set at 30 sccm. For Cu_4_O_3_ deposition, the working pressure was 1.51 Pa and the gas flow was set at 27 sccm of O_2_ and 3 sccm of Ar. CuO samples were deposited with thicknesses of 30 nm, 55 nm, and 130 nm. Cu_4_O_3_ films were deposited with thicknesses of 35 nm, 55 nm, and 115 nm.

### Characterisation

The samples were characterised with XRD and SE to study their structural and optical properties. We used the PANalytical X’Pert PRO diffractometer, with a Cu anode (0.154 nm radiation wavelength) for diffraction measurements. Ellipsometric measurements were performed with J.A. Woollam M 2000 ellipsometer. The thickness of thin films was measured with a Talystep profilometer.

### Ion implantation

Thin film samples were subjected to ion implantation with Cr ions at the Henryk Niewodniczanski Institute of Nuclear Physics in Krakow. The ion implantation equipment features a modified Bernas-type source of ions and electromagnets that precisely select the ions with desired energy and mass. As a source of Cr ions we used anhydrous chromium trichloride. The energy and dose of implanted ions were 15 keV and 5 × 10^16^ ion/cm^2^. The dose of ions was controlled by the time that a moving sample holder spent in front of the ion beam.

The implantation of Cr ions into copper oxides was simulated with the Stopping and Range of Ions in Matter software. For visualization of recoils 1000 ions were simulated, for other results 30,000 ions were simulated.

## Results and discussion

### Implantation simulations

**Figure 1:**
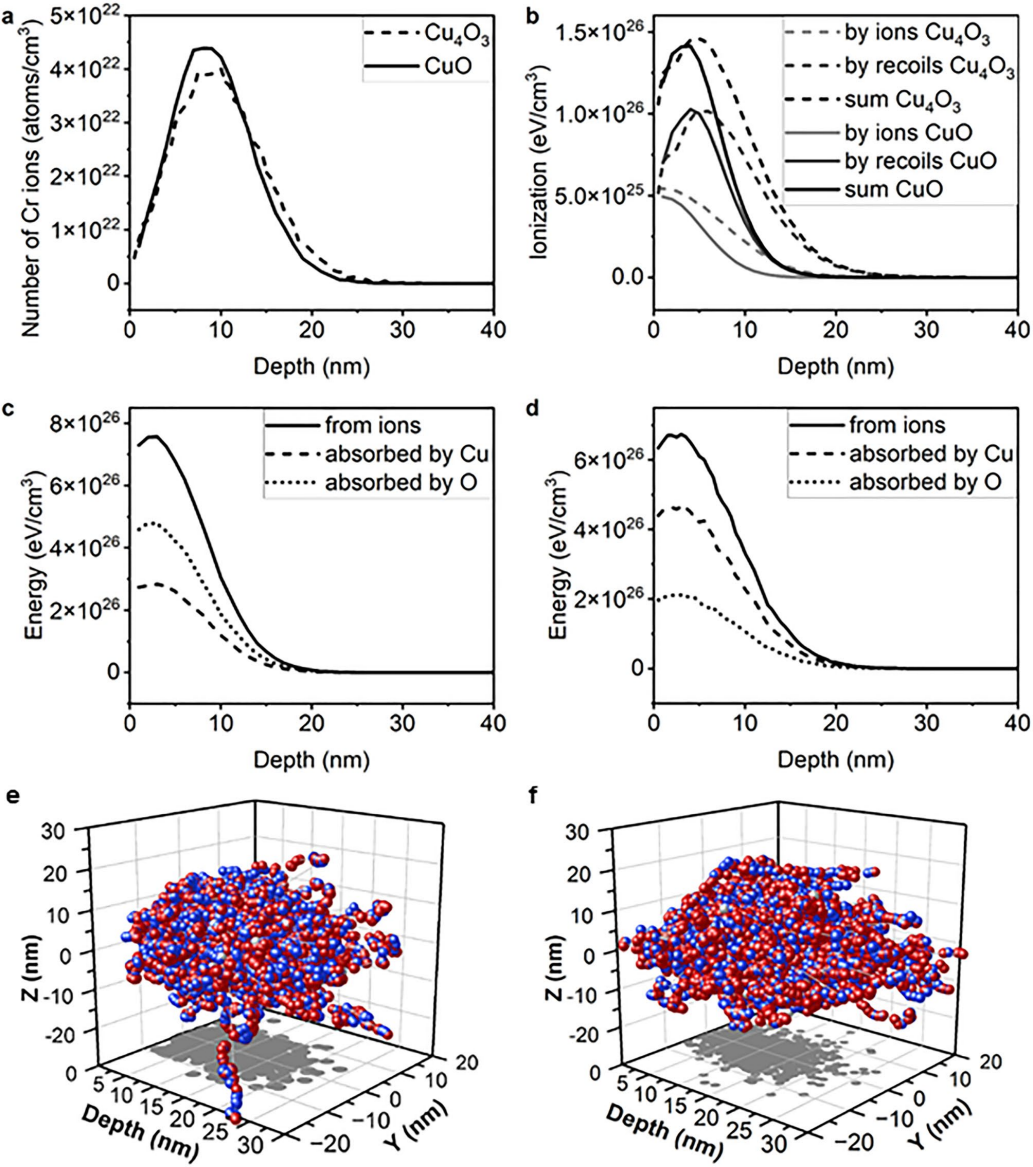
(**a**) Depth distribution of Cr ions implanted into copper oxides with energy of 15 keV and dose 5 × 10^16^ cm^−2^, (**b**) ionization of target atoms caused by ions and recoils, (**c**) energy brought by ions and its absorption by Cu and O atoms in implanted CuO and (**d**) Cu_4_O_3_, (**e**) recoil cascades for implantation of CuO (the balls represent atoms: grey—Cr ions, red—Cu, blue—O; an arrow indicates the beam incidence point; on XY plane a grey projection of Cr ions positions is presented (**f**) recoil cascades for implantation of Cu_4_O_3_.

SRIM simulations can provide information about the interaction of implanted ions and target atoms. Basic information is the distribution of implanted atoms, which depends on their energy, and the density and composition of the target. Therefore, for each copper oxide, the distribution of Cr ions will be different. The distribution of ions, and therefore their range, for CuO and Cu_4_O_3_ implanted with 15 keV energy Cr ions are presented in Fig. 1a. The oxides CuO and Cu_4_O_3_ have density of 6.315 g/cm^3^ and 5.84 g/cm^3^, respectively. The difference in the ion range is not large, the composition of the oxides plays an important role here, Cu_4_O_3_ has a higher ratio of Cu with respect to O than CuO, and the two elements have different displacement energies, which is the main cause of the difference in the ion depth distribution. Ionisation is in a greater share caused by recoils than ions, as presented in Fig. 1b. Cu_4_O_3_ becomes more ionised than CuO when implanted with the same energy and dose of ions. The energy introduced by Cr ions can be absorbed by O or Cu atoms (Fig. 1d,e). In the case of Cu_4_O_3_, the Cu atoms absorb more energy than the O atoms (Fig. 1b), for CuO the absorption share is inversed (Fig. 1a), this is caused by differences in oxides composition. Energy absorption occurs mainly near the surface of the target and decreases in depth. The increase in the relative amount of the Cu atoms to the O atoms is responsible for a larger ionisation depth in Cu_4_O_3_ oxide.

The SRIM package allows us to illustrate the recoil cascades and see how much more damage is spread by the recoils than just ions, also in the lateral direction to the beam. The simulation was carried out for ions with a 0° angle of incidence, where the beam enters the target at a single point. To calculate the data in Fig. 1a–d, 30,000 ions were simulated, while for the graphical presentation in Fig. 1e,f only 1000 ions were simulated. Each grey ball represents a Cr ion, and red and blue balls represent copper and oxygen atoms, respectively. An energised ion knocks the target atoms of their positions, and if an atom has enough energy, it knocks another one, creating recoil cascades. This effect is more profound in the case of Cu_4_O_3_. The trajectories of 1000 simulated ions extend to a greater extent in directions perpendicular to the beam for Cu_4_O_3_ (Fig. 1f) than CuO (Fig. 1e). It is important to emphasize that the results presented in Fig. 1e,f show the effect of only 1000 ions that enter the material at a single point (it is impossible to make graphical visualisations for larger amount of ions), while the real implantation experiment is subjected to the entire surface of the sample and the number of ions is drastically larger with a dose of 5 × 10^16^ cm^−2^.

### Structural properties—XRD

**Figure 2:**
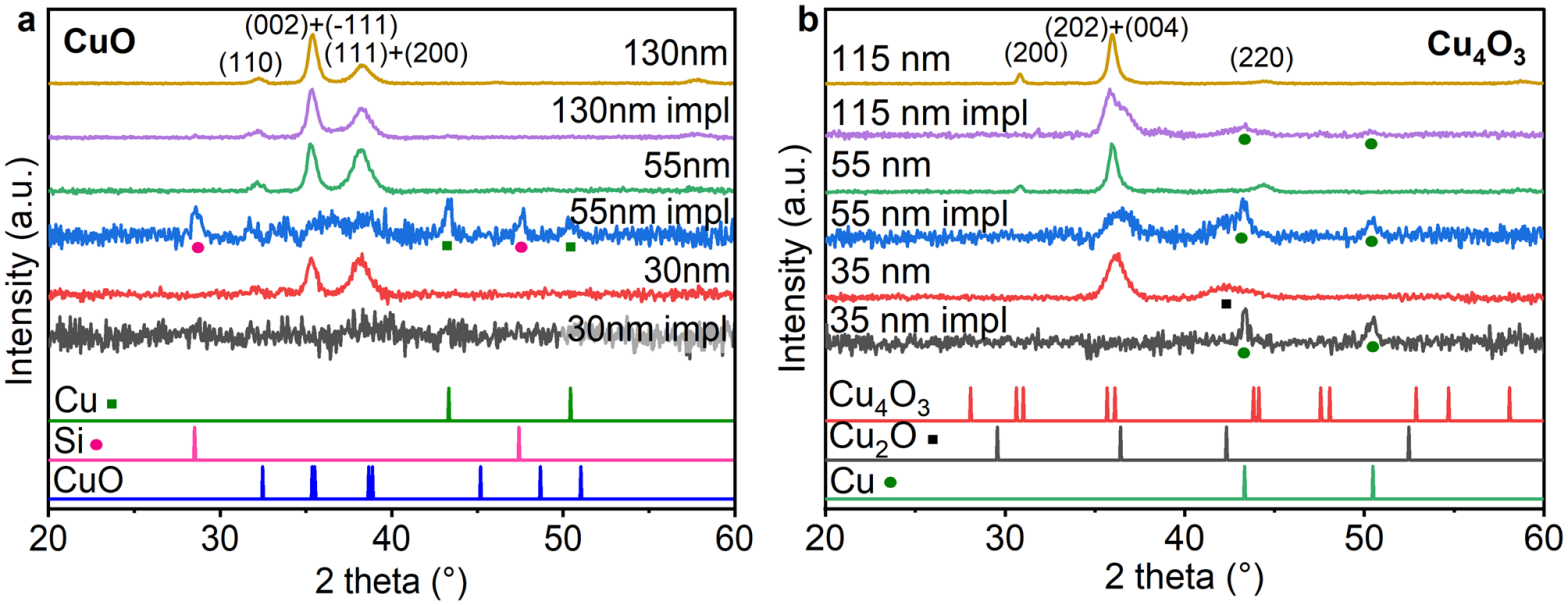
Diffractograms of thin films before and after implantation with Cr ions (**a**) CuO, (**b**) Cu_4_O_3_; peaks for CuO and Cu_4_O_3_ are designated.

XRD measurements were performed to study how implantation changed the phase composition and crystallinity of the thin film copper oxides, as well as to confirm if the correct oxide was deposited. More detailed analysis, including the calculation of lattice strain and crystallite size, of Cr-implanted CuO thin films can be found in^28^ (Fig. 2a). In Fig. 2a diffractograms of samples of 30 nm, 55 nm, and 130 nm are presented, all of them show clear CuO peaks (based on JPCDS card #01-080-0076). In the case of a 30 nm layer, the implantation completely amorphized the material, there are no visible peaks after implantation. The 55 nm sample after implantation shows peaks from Cu (JPCDS card #00-001-1242) and Si (JPCDS card #00-001-0787), while no clear peaks from CuO are visible. The thicker sample still shows CuO peaks after implantation. The ions implanted in the material lose their energy via excitation of target’s electrons or collisions with nuclei. For energies in the range of keV, the nuclear stopping is predominant^26^. Collisions with target atoms cause them to move from their positions in the crystal lattice, the crystal structure is damaged and can be amorphized with enough implanted ions^21^. A portion of the target atoms can also be sputtered from the target, which could also be a reason for the absence of a signal from the implanted 30 nm sample. From this information, we can consider that the ions influence the crystal structure drastically up to 55 nm in depth, but a thicker layer is not damaged and still retains its crystal structure beyond the range of ion implantation. No Cr compounds were identified using the XRD method; one explanation is that no crystalline structures were formed by Cr, and another is that their amount was too little to be detected with this method.

The thinnest Cu_4_O_3_ sample shows a peak coming from Cu_2_O (JPCDS card #01-074-1230), indicating that deposition was not completely successful (Fig. 2b). For thicker samples, 115 nm and 55 nm, the film consists only of Cu_4_O_3_ with peaks visible: (200), (202) and (404), (220)—JPCDS card #00-0491-830. After implantation, Cu peaks are visible (JPCDS card #00-001-1242). In the case of 55 nm sample, the crystal structure was deteriorated to such degree that the paramelaconite peaks are barely visible. The thin film was damaged by implantation. The thinnest sample is the most damaged as the ions and recoils influenced its entire thickness, and according to the simulation results, the range of recoil is about 30 nm. The 55 nm sample is heavily damaged as the recoils reach further in depth of material, and influence most of the sample. Therefore, to study optical properties with SE we chose only Cu_4_O_3_ with a thickness of 115 nm.

For samples with thickness higher than 100 nm, the implantation had a more profound effect on Cu_4_O_3_ than CuO. This corresponds to the results of the SRIM simulations where the ionisation and impact on target atoms are greater for Cu_4_O_3_ (Fig. 1). In case of thinner samples, CuO crystal structure was more damaged, and for the thinnest sample no crystalline material was detected, this observation may be caused by difference in thickness between the samples CuO and Cu_4_O_3_ samples (Cu_4_O_3_ is slightly thicker).

### Optical properties—SE

**Figure 3:**
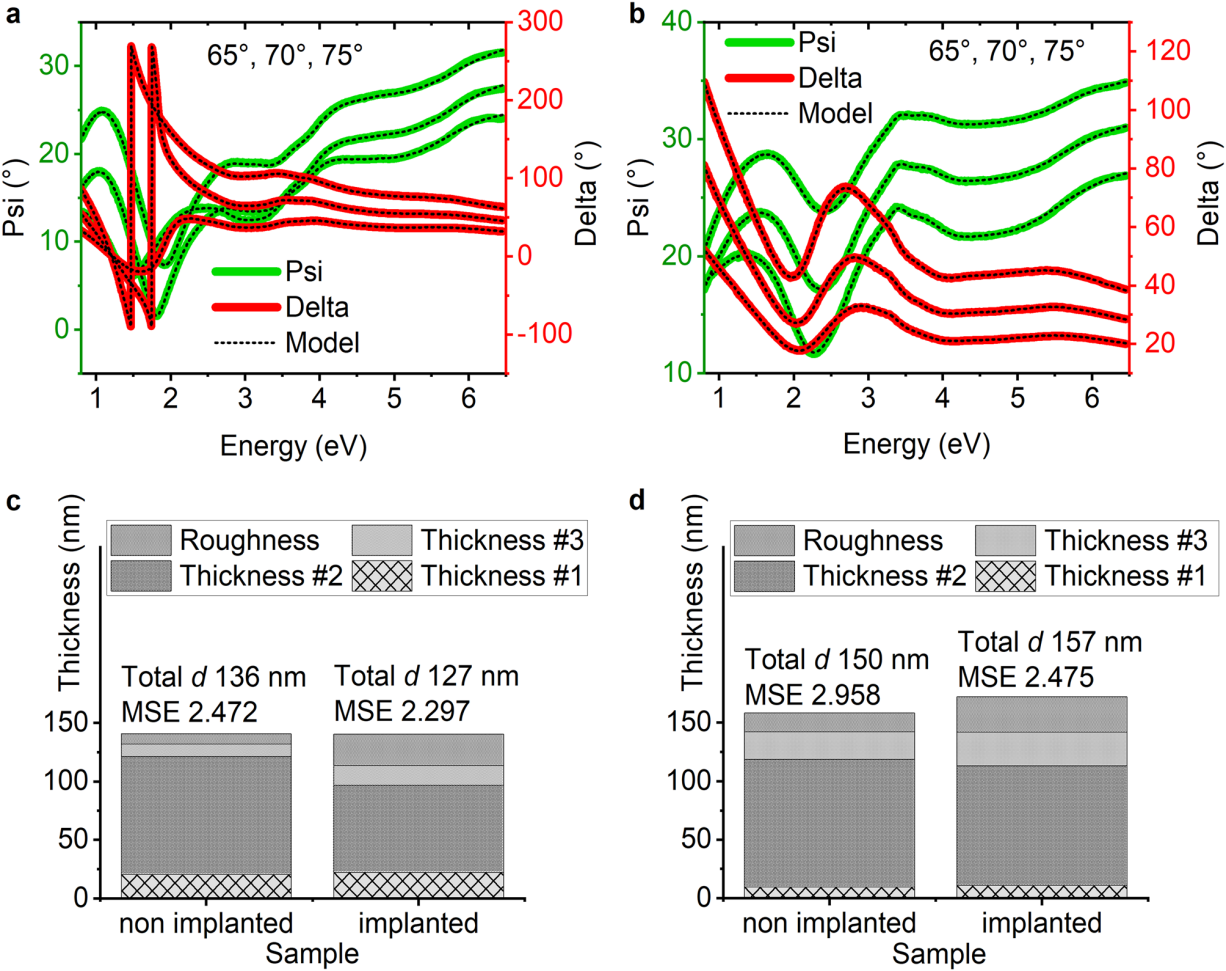
SE measured Psi and Delta data and fitted model values for Cu_4_O_3_ sample (**a**) nonimplanted, (**b**) implanted with Cr ions, SE model results after fitting for (**c**) CuO, (**d**) Cu_4_O_3_; thickness #1 represents interface between deposited layer and the substrate, thickness #2 is the main layer of thin films, thickness #3 with roughness are layers that represent influence of implantation.

Spectroscopic ellipsometry is a method that is used to study thin film samples, especially. In this technique, a polarised light beam is directed onto the sample and after reflection gets into the detector. The change in light polarisation is the result of SE measurement and is expressed as two parameters: Psi and Delta, which give information on the amplitude and phase changes, respectively. The measured data along with the fitted values are presented in Fig. 3a,b for the non-implanted and implanted Cu_4_O_3_ samples.

To analyse the data, an optical model of the desired material must be used. Here, we establish models for CuO and Cu_4_O_3_ based on information about energy transitions from^4^ which is presented in Table 1 together with fitted values of the oscillators’ energy positions E_0_. We used Tauc–Lorentz oscillators to model each energy transition and multiple oscillators to model the dielectric function. The T-L oscillator is expressed as:

1 $$\begin{gathered} \varepsilon_{T - L} \left( E \right) = \varepsilon_{1} - i\varepsilon_{2} \hfill \\ \varepsilon_{2} = \left[ {\frac{{Amp E_{0} Br\left( {E - E_{g} } \right)^{2} }}{{\left( {E^{2} - E_{0}^{2} } \right)^{2} + Br^{2} E^{2} }} \cdot \frac{1}{E}} \right], E > E_{g} \hfill \\ \varepsilon_{2} = 0, E \le E_{g} \hfill \\ \varepsilon_{1} = \frac{2}{\pi }P\mathop \smallint \limits_{{E_{g} }}^{\infty } \frac{{\xi^{\prime}\varepsilon_{2} \left( \xi \right)}}{{\xi^{2} - E^{2} }}d\xi \hfill \\ \end{gathered}$$

where ε_1_—real part of dielectric function, ε_2_—imaginary part of dielectric function, E_g_—energy band gap, E—photon energy, E_0_—peak central energy, Amp—amplitude, Br—broadening, P—Cauchy principal value.

**Table 1:** Transition energies of CuO and Cu_4_O_3_ from^4^ and fitted Tauc*–*Lorentz oscillators position energies E_0_.

CuO			Cu_4_O_3_		
Transition energies [eV]	T–L oscillator positions [eV]		Transition energies [eV]	T–L oscillator positions [eV]	
	non implanted	implanted		non implanted	implanted
1.66	0.84	1.08	1.80	0.52	0.38
2.07	2.40	2.16	2.34	1.96	1.92
2.68	3.28	3.29	3.08	2.87	2.70
3.46	6.09	5.70	3.53	3.37	3.26
			3.90	3.80	3.71
			5.29	3.84	3.75
			5.68	5.69	6.00

The model for CuO consists of a Si substrate, three layers of CuO, and a roughness layer. Each CuO layer consists of 4 Tauc–Lorentz oscillators. The roughness layer is a volume sum of 50% void and 50% of the layer below. Therefore, to calculate the total thickness of the film, we need to add the thicknesses of three oxide layers and half of the roughness layer. The Cu_4_O_3_ model is similar, the only difference being that the oxide layers consist of seven Tauc–Lorentz oscillators. Although in^4^ five transitions are assigned to CuO, our model uses only four oscillators, because adding the fifth one did not improve the fitting. The resulting fitted thicknesses and the mean squared error obtained are presented in Fig. 3c,d. The roughness and top layer are the parts of the films that were influenced by implantation. The sum of their thicknesses (half of the roughness layer) increases with implantation, as the ions change the material properties in the depth of the sample. According to the SRIM simulation this depth is about 30 nm. The SE results show that the sum of layers R and #3 is about 30 nm for Cu_4_O_3_, 40 nm for CuO implanted with 15 keV energy ions.

**Figure 4:**
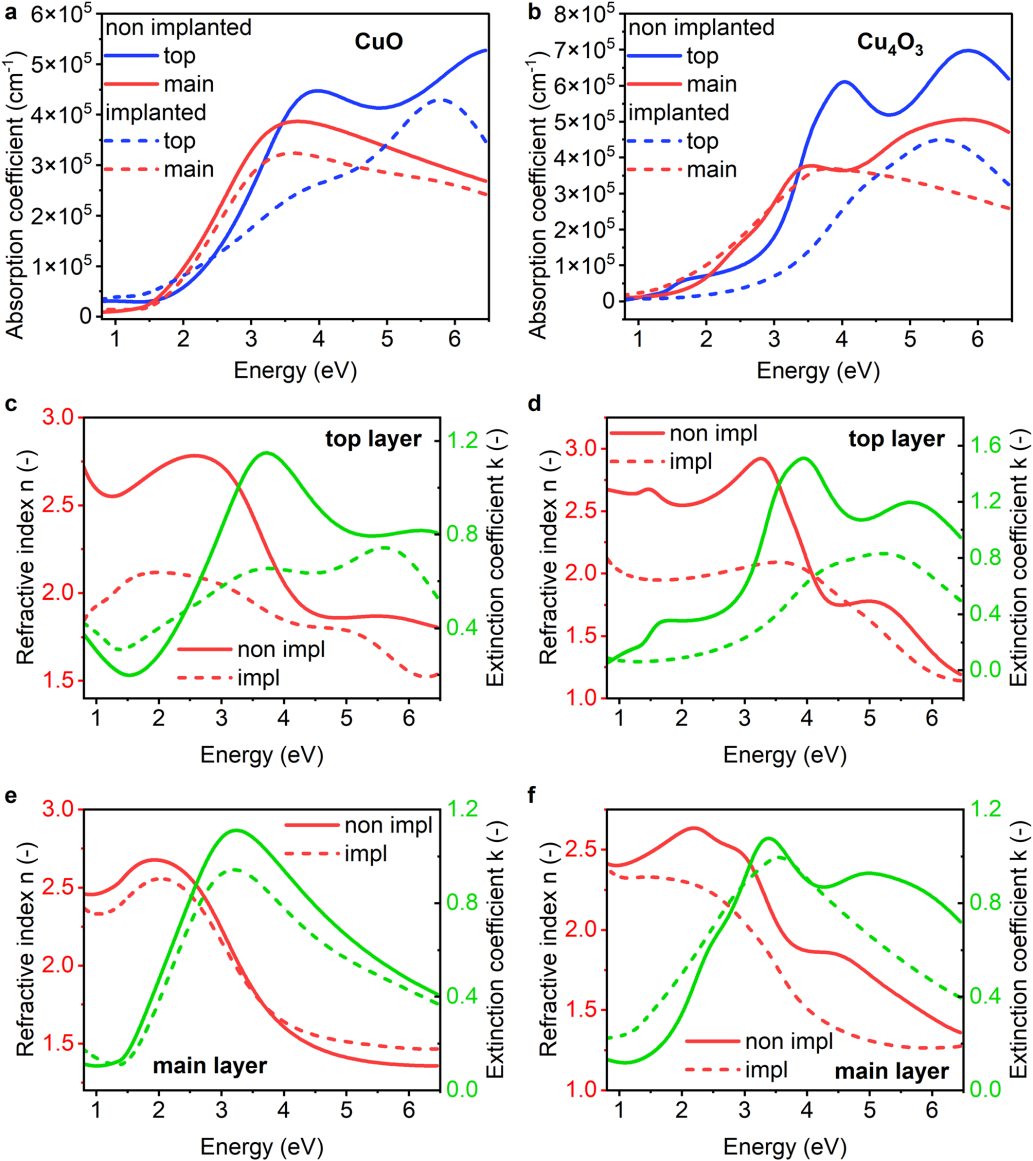
Optical properties of copper oxides: absorption coefficient of CuO top and main layers (**a**) and Cu_4_O_3_ (**b**), refractive index and extinction coefficient of top layer of CuO (**c**) and Cu_4_O_3_ (**d**), and for main layers of CuO (**e**) and Cu_4_O_3_ (**f**).

The roughness layer is thicker for implanted samples than for nonimplanted samples in each case. The total thickness of the films is calculated as different than what was desired during the deposition process. It could be caused by nonidentical deposition conditions or limitations of the SE method which is an indirect one. The bottom layer’s function in the model is to simulate the interface between the substrate and deposited oxide, therefore, the optical properties of this layer are not considered in the analysis of the optical properties of copper oxides. The layers in which we are interested are the top layer (#3) and the main layer (#2). In Fig. 4 the absorption coefficient, refractive index, and extinction coefficient of the studied samples are presented. In case of both oxides, the implantation reduced the absorption of material especially for energies higher than 3 eV. The optical parameters n and k also have lower values in most of the energy range after implantation.

In Fig. 4a we can see that CuO main layer absorption decreased and the energy band gap would increase. The slope of the absorption coefficient of the top layer is strongly reduced after implantation, which would indicate an increased Urbach energy and absorption by defects. For Cu_4_O_3_, we can observe this increase of Urbach energy more for the main layer (Fig. 4b). In the case of Cu_4_O_3_, the top layer is the source of more light absorption, both before and after implantation. The n and k characteristics show that the top layer has more profound oscillators in the fitted model. These two parameters also have higher values in the case of the top layer than in those for the main layer. Compared to the literature^29^, n and k are agreeable, the value n reaches a maximum of approximately 2.5–3 and decreases towards energies higher than 3 eV; k increases toward higher energies. The top layer has lowered its extinction coefficient after implantation to a greater extent than the main layer; this shows that the implantation process has a greater influence on the top of the sample. The absorption coefficient of CuO decreases after implantation, and the properties of the top layer changed more distinguishably than those of the main layer (Fig. 4a,c,e). The refractive index value falls from 2.5 to 1.5 with increasing energy (Fig. 4e), which is consistent with the literature^30^. Both n and k almost do not change for the main layer after implantation, while for the top layer their values decrease significantly, n from maximum 2.5–2.2, and k from maximum almost 1.2 to less than 0.8, and the maximum value of k shifts to higher energies. For both oxides, the drop in absorption origins mainly in decrease of amplitude of oscillators with energy positions of about 3.5 eV.

## Conclusions

In this work, we deposited thin films of CuO and Cu_4_O_3_ that were subjected to implantation with Cr ions. Simulations of the implantation process allowed us to assess the range of sample depth in which implantation has a significant impact. XRD studies showed that samples with a thickness close to the ion penetration range were largely damaged, their crystallinity decreased, and phase changes occurred. The three-layer model based on Tauc–Lorentz oscillators gave a good fit with a mean squared error value less than 3. The determined values of the oscillator positions are close to the energy transition energies from the literature. The use of the three-layer model indicated that the change in the optical properties of the oxides came from a maximum layer depth of 45 nm, which is consistent with the results of the SRIM simulation. The calculated absorption coefficient is lower for implanted samples. Reducing the absorption of oxides is not a desirable effect for application as an absorber layer in a solar cell. Annealing and recrystallisation of the layers, leading to the distribution of the dopant throughout the volume of the layers, may result in a different effect, especially in the modification of the electrical properties of copper oxides. Thus, the next step of this research is to study thin films after annealing.

## Data Availability

Data is available on request from the corresponding author.
